# Detection of Glyphosate in Drinking Water: A Fast and Direct Detection Method without Sample Pretreatment

**DOI:** 10.3390/s18092961

**Published:** 2018-09-05

**Authors:** Jafar Safaa Noori, Maria Dimaki, John Mortensen, Winnie E. Svendsen

**Affiliations:** 1 Department of Micro- and Nanotechnology, Technical University of Denmark, 2800 Kgs. Lyngby, Denmark; Maria.Dimaki@nanotech.dtu.dk (M.D.); Winnie.Svendsen@nanotech.dtu.dk (W.E.S.); 2 IPM—Intelligent Pollutant Monitoring ApS, 2690 Karlslunde, Denmark; 3 Department of Science and Environment, Roskilde University, 4000 Roskilde, Denmark; john@ruc.dk

**Keywords:** pesticide, glyphosate, water, electrochemistry, sensors

## Abstract

Glyphosate (Gly) is one of the most problematic pesticides that repeatedly appears in drinking water. Continuous on-site detection of Gly in water supplies can provide an early warning in incidents of contamination, before the pesticide reaches the drinking water. Here, we report the first direct detection of Gly in tap water with electrochemical sensing. Gold working electrodes were used to detect the pesticide in spiked tap water without any supporting electrolyte, sample pretreatment or electrode modifications. Amperometric measurements were used to quantify Gly to a limit of detection of 2 μM, which is below the regulation limit of permitted contamination of drinking water in the United States. The quantification of Gly was linearly proportional with the measured signal. The selectivity of this method was evaluated by applying the same technique on a Gly Metabolite, AMPA, and on another pesticide, omethoate, with a chemical structure similar to Gly. The testing revealed no interfering electrochemical activity at the potential range used for Gly detection. The simple detection of Gly presented in this work may lead to direct on-site monitoring of Gly contamination at drinking water sources.

## 1. Introduction

Glyphosate is one of the most commonly used pesticides in the world as it can target a wide range of plants. Although early research on glyphosate (Gly) has reported a low toxic impact on humans, recent studies suggest that Gly-based pesticides can affect cell cycle regulations in both plants and animals [1,2,3,4]. The intensive use of Gly has led to the appearance of transgenic plants that are resistant to Gly. Evidence has demonstrated that even low concentrations of Gly may lead to the stimulation of hormone-dependent cancers in humans [5].

The continuous use of Gly has raised concern regarding residue appearance in ground water [3,6]. Pesticide concentrations are strictly regulated in ground water, and consequently in drinking water. In the United States, the permissible limit for pesticide concentration in drinking water is 4 μM and in the EU the limit is 0.6 nM [3]. Today, the regulation limits are monitored by highly difficult and costly chromatographic techniques. Due to the demanding sampling procedure, only a few samples are collected on a yearly basis. There is a need for the development of an affordable, rapid and sensitive analytical method that can frequently identify and quantify Gly on-site. The movement of a pesticide from the surface to the ground water table is dependent on the soil properties. The need for frequent measurements is relevant at sandy soil profiles, for example, since the contamination can move relatively quickly towards a ground water table. The fast movement of contamination can be reinforced with heavy rains, resulting in undetected contamination events between the yearly mandatory sampling. Continuous or frequent monitoring of water contamination can provide an alert in the case of contamination.

Electrochemical sensing offers the possibility of rapid, on-site measurements of contaminants in drinking water. In the past few years, several studies have started reporting that electrochemical measurements will be the future solution for fast and accurate measuring of contaminants in water [7,8]. Developments in the electronics and electronic communication fields has led to the rethinking of the way we are conducting field measurements. It has presented the possibility of moving electrochemical measuring techniques from the laboratories into the field [8]. Thus, the main challenge with on-site electrochemical pollution monitoring is to develop electrochemical detection procedures for targeted contaminants. With automatic electrochemical sensing, frequent monitoring of water quality can be carried out at water utilities at a considerably reduced cost. 

Glyphosate has been reported as a non-electroactive compound that cannot be measured at accessible potentials [6]. Therefore, earlier studies have only been able to electrochemically detect Gly using complicated multistep procedures, electrode material modifications and sample treatments. Previous studies have used a hanging mercury drop as a working electrode for detecting Gly [9,10]. The use of electrode surface modification is another common approach for detecting Gly in electrolytes. Electrochemical pretreatment of electrodes, combined with sample pretreatment, has been reported to be a selective method for Gly detection [3,11]. All of these methods are not applicable in the field, as they require sample pretreatment or liquid electrodes that could introduce contamination to the water themselves.

In this study, we report an electrochemical measuring method for Gly detection using unmodified electrodes. The electrochemical detection is based on the oxidation of Gly on a gold electrode surface. To the best of our knowledge, this is the first time Gly has been directly detected in water without any electrode modification or sample treatment. 

## 2. Materials and Methods

### 2.1. Materials

Glyphosate was purchased from Sigma Aldrich Chemie GmbH, 45521, Buchs, Switzerland. Dilution series of up to 0.3 mM were prepared in local tap water (Lyngby Taarbæk municipality, Denmark) with no further treatment or purification. Water characterization can be seen in the Supplementary Material, Table S1. Tap water was also considered as the background in all measurements. Omethoate was purchased from Sigma Aldrich, 36181. Aminomethylphosphonic acid (APMA) was purchased from Sigma Aldrich, 324817. A concentration of 0.3 mM was prepared using local tap water with no further treatment or purification. All electrochemical measurements were taken with a potentiostat (PalmSens 3, PalmSens, Houten, The Netherlands). Commercial, screen-printed electrodes based on gold working and counter electrodes and a silver reference electrode were used to conduct the measurements (DRP 220AT, Dropsens, Asturias, Spain). The pH measurements were carried out using a pH meter (EUTECH Instruments pH700, Singapore). The conductivity of the water was measured using a conductivity meter (Meter Lab CDM210, Radiometer Copenhagen, Lyon, France).

### 2.2. Methods

Different Gly concentrations were prepared in local tap water. The conductivity of the water was measured to be 275.4 μS/cm. The pH of the prepared Gly solution was monitored at the different concentrations showing an average pH value of 8.05 ± 0.1.

An electrochemical profile for Gly was obtained by cyclic voltammetry (CV) using a potential range of 0.0–1.0 V and a scan rate of 0.05 V/s. The starting potential for this experiment was determined after measuring the open circuit potential, which was 0.0228 V. Three cycles were measured and the first cycle was considered for the final evaluation. A fresh, unused sensor was used for each measurement and then disposed. All sensors were used without cleaning [12]. Square wave voltammetry (SWV) was also used in the potential range of 0.4 to 1.0 V with a frequency of 10 Hz and step potential of 0.01 V to confirm the peak potential. 

The optimal potential for quantification of Gly was found by amperometrically testing concentrations of 0.3 mM and 0.018 mM of Gly and in tap water as a background signal. The tests were carried out at 7 different potentials extracted from the obtained CV and SWV measurements. Amperometric measurements were conducted to quantify increasing Gly concentrations at this optimal potential (0.78 V) for 50 s. Three replicates were performed for each concentration from the independent dilution series. Data were collected and processed using PSTrace 5 version 5.4. Peaks were manually selected using a built-in function that identifies the peak from user input of the start, peak and end location. The signal was integrated between 0 and 50 s to obtain the charge corresponding to each concentration. 

Measurements on unprocessed water were conducted during this study. Ground water was obtained from local water producers “Gevninge Vandværk”. The water was collected directly after pumping it out from the well and used with no further treatment. When stored, the sample was kept in a dark fridge at 4 °C in a closed container to prevent contact with oxygen. The ground water was spiked with Gly, reaching a concentration of 0.3 mM. The electrochemical profiles of the un-spiked ground water and the spiked ground water were recorded using cyclic voltammetry. One cycle was conducted starting from the open circuit potential of 0.015 V, with a scan rate of 0.05 V/s. Only one cycle was recorded, as Gly is an irreversible compound. 

All measurements were conducted by covering the electrodes of the sensor with 70 μL of sample. A fresh sensor was used for each measurement to avoid cross contamination.

## 3. Results

### 3.1. Electrochemical Response of Glyphosate

**Figure 1:**
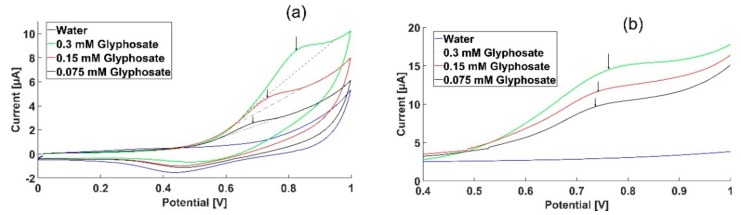
Electrochemical profiles of glyphosate and local tap water. (**a**) Cyclic voltammogram of 0.3, 0.15 and 0.075 mM of glyphosate (green, red and black respectively) and tap water (blue), using gold working electrodes; (**b**) Square wave voltammetry measurements of 0.3, 0.15, 0.075 mM of glyphosate (green, red and black, respectively) and water (blue), using gold working electrodes. The observed peak potential for glyphosate is 0.79 V.

To identify suitable electrode materials for Gly detection, different electrode materials were screened, such as carbon, chromium, bismuth and gold. The screening showed that a Gly response was only obtainable from gold electrodes and it was therefore decided to continue with gold electrodes for Gly quantification. To identify the potential at which the interaction between Gly and the electrode surface occurs, we screened three different concentrations of Gly, 0.3, 0.15 and 0.075 mM with CV, as seen in Figure 1a. A peak (indicated by the arrow) can be observed at 0.79 V in the CV of 0.3 mM Gly. No peak was observed in the cyclic voltammogram of the water background. When decreasing the Gly concentration, the peaks are shifted towards lower potentials. The increasing peak potential with increasing concentration can be due to the saturation of the electrode surface, that slows the kinetics of the reaction at a higher concentration. To verify this peak potential, SWV was used. Square wave voltammetry is a more sensitive technique than CV as it minimizes non-faradaic currents. The measured current was plotted against the applied potentials as seen in Figure 1b. A more prominent peak was observed between 0.6 V and 1 V using SWV compared to CV, confirming that an electrochemical reaction occurs for Gly that is distinguishable from the background signal. Figure 1a,b clearly shows that the peak current response in both CVs and SWVs is proportional to the change in Gly concentration, which confirms that the obtained response is from Gly. The results shown in Figure 1a,b suggest that potential optimization is necessary in order to obtain the highest current response.

### 3.2. Selecting Optimal Potential for Glyphosate Quantification

**Figure 2:**
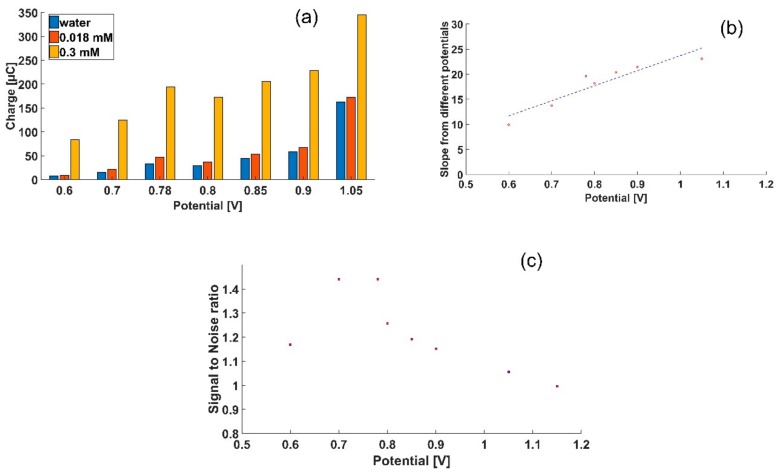
Optimization of the detection potential for glyphosate. (**a**) Charge extracted from amperometry measurements of glyphosate and tap water conducted at different potentials; (**b**) The slopes extracted from (**a**) as a function of potential; (**c**) The signal to noise ratio at the concentration of 0.018 mM as a function of potential.

We mapped the contribution of the water background, to the glyphosate signal, by screening the current output in a narrow potential range around the target potential found using CV and SWV. Seven potentials were selected and amperometric measurements were carried out for each potential, on Gly concentrations of 0.3 mM, 0.018 mM and the water background. The charge was extracted by integrating the amperometric measurements. Figure 2a shows the charge obtained from the amperometric measurements conducted at the seven selected potentials within the range of 0.6–1.05 V.

To find the optimal potential to carry out the quantification of Gly, the slopes of the charge detected at the various potentials were calculated, based on the measured Gly concentrations. The slope represents the sensitivity that can be obtained from each potential. Figure 2b presents the sensitivities plotted against the potential. The steeper the slope, the higher the sensitivity. The signal-to-noise ratio (S/N) is here determined by the measured charge of 0.3 mM, divided by the measured charge of the background. The S/N for the concentration of 0.018 mM as a function of potential is shown in Figure 2c. Ideally, a high sensitivity and a high S/N is preferred for sensors. The optimal sensitivity can be achieved at the highest tested potential (1.05 V). However, the S/N at this potential was the worst (Figure 2b,c). The highest S/N ratio was found in the potential range of 0.7–0.78 V. Given the higher sensitivity measured at 0.78 V, this potential was selected as the optimal potential for further quantification of Gly. 

### 3.3. Amperometric Quantification of Glyphosate

**Figure 3:**
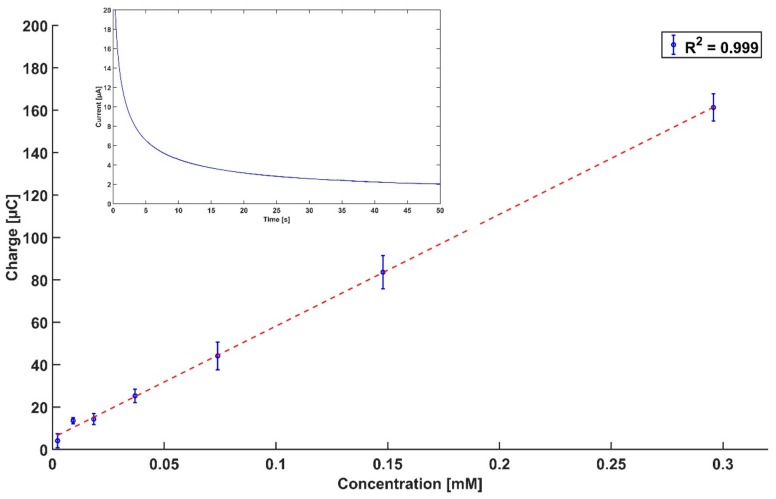
Charge as a function of concentration calculated by integrating the measured current at a time interval of 50 s (*n* = 3, replicates from independent dilution series). The inset shows the amperometric measurement of 0.3 mM of Gly; the full dataset is presented in Supplementary Material, Figure S1.

A dilution series of Gly was quantified by amperometric measurements at 0.78 V for 50 s. The signal stabilized at higher currents with increasing Gly concentration. The charge corresponding to each concentration was found by integrating the measured current (inset of Figure 3). The obtained charges were plotted against the respective concentrations, as seen in Figure 3. A linear relationship between the charge and the concentrations was found with an R^2^ of 0.999, indicating an excellent linear fit. The water background signal was subtracted from the results in Figure 3. The detection limit of this method was theoretically calculated to be 1.6 μM, based on three times the standard deviation of the background water signal, divided by the slope of the calibration curve.

### 3.4. Preliminary Selectivity Test

**Figure 4:**
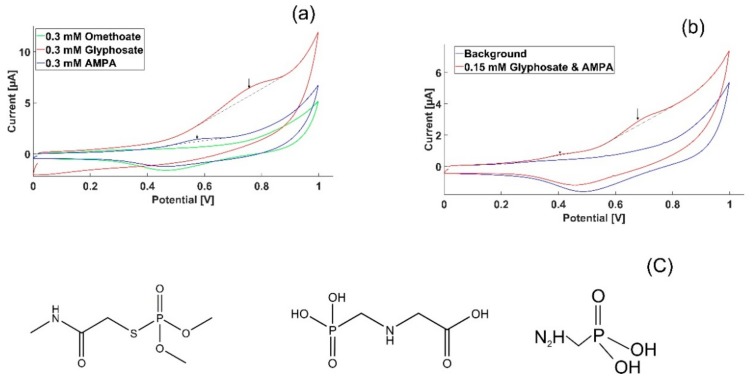
Interference signal from different compounds with a similar structure to Gly. (**a**) Cyclic voltammetry of glyphosate, AMPA and omethoate using a gold working electrode. Glyphosate shows a distinct peak around a potential of 0.78 V, while no electrochemical activity appears in this potential range for omethoate. AMPA is electrochemically responding at a potential of 0.57 V and is well separated from the Gly response; (**b**) Cyclic voltammetry profile of one sample containing both Gly and AMPA with a concentration of 0.15 mM each, showing clear peak separation corresponding to the two compounds; (**c**) Chemical structures for omethoate, glyphosate and AMPA, respectively.

To challenge the selectivity of the sensor to glyphosate, a signal interference test was conducted on AMPA, a metabolite of Gly always found in water where glyphosate is present, and omethoate, a widely used pesticide in the EU. Both chemicals have a similar chemical structure to Gly and AMPA is the chemical most likely to interfere with the measurements. Glyphosate, AMPA and omethoate were separately tested using the same concentration of 0.3 mM, respectively. Figure 4a shows that there is no interference from AMPA and omethoate at the characteristic potential of Gly found in this work. A small response from AMPA was observed at a potential of 0.57 V, and it was well separated from the Gly peak response at 0.79 V. The profile of a mixed sample containing a total concentration of 0.15 mM of Gly and 0.15 mM of AMPA was measured by cyclic voltammetry using the parameters earlier described, as seen in Figure 4b. A clear peak separation is seen between the two mixed compounds as the Gly peak response appears at 0.75 V, while AMPA shows a peak at 0.57 V.

### 3.5. Real Sample Test

**Figure 5:**
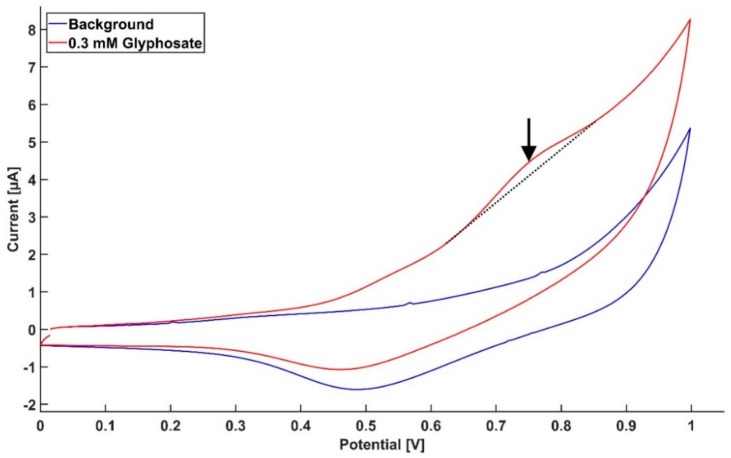
Cyclic voltammogram profile for a ground water sample spiked with Gly, with a final concentration of 0.3 mM. The graph shows a clear response from Gly compared to the un-spiked sample at the expected potential range. The sample was used directly after extraction with no further treatment.

The applicability of this method was tested on a real ground water sample. The sample was spiked with Gly to reach a 0.3 mM concentration. It can be seen in Figure 5 that there is a clear response from Gly, when compared to the un-spiked sample. It should be noted that the sample was collected, and tested, without pretreatment or addition of any electrolyte. 

Differences in salts and ionic species in ground water, lake water or tap water could result in differences in the electrode response. It is therefore necessary for future field applications to calibrate the standard curve according to the target water from where measurements will be conducted.

## 4. Discussion

Cyclic voltammograms of glyphosate conducted with gold working electrodes have revealed that oxidation occurs at 0.78 V. Earlier studies have reported that a strong interaction occurs between phosphorous and gold [13,14,15,16]. A study that aimed to detect the total phosphorus concentration in water showed a three-fold signal enhancement when introducing gold nanoparticles to the working electrode surface [17]. Looking at the chemical structure of glyphosate, as seen in Figure 4c, we postulate that oxidation might occur at the phosphorous covalent bond with oxygen. In order to achieve a redox reaction around this strong bond, a relatively high potential is needed. When the covalent bond breaks, the phosphorus will have a free electron that can bind strongly to the gold electrode. This could explain why the glyphosate oxidation was measured at a relatively high potential. 

Phosphorus exists in other compounds and is a major cause of water contamination and environmental eutrophication. The existence of phosphorus and other pollutants in water may raise concerns regarding signal interference from different compounds and the uniqueness of the obtained signal towards glyphosate when measuring directly in the field. The signal interference from other compounds containing phosphorus will have standard potentials that differ from the signal obtained from the Gly redox reaction. The phosphorus bonds formed in other compounds will need different energies to obtain a redox reaction and, consequently, the standard potential will differ. We tested this hypothesis by investigating omethoate, another pesticide with a chemical structure close to Gly, and AMPA, a metabolite of Gly; indeed, no overlapping electrochemical activity with Gly was observed; both of these compounds have phosphorus bonds, but to different elements, which strengthens our hypothesis. A more exhaustive list of possible interfering compounds needs to be tested in order to establish the uniqueness of the proofed potential for Gly identification, but our data so far suggests that this method is a promising candidate for selective Gly quantification in the field.

As this study is an attempt at direct field testing, it was a requirement to have no sample pretreatment. Electrolyte is normally added to overcome technical challenges by increasing the conductivity of the solution and minimizing the resistance between the working and counter electrode [18]. The absence of electrolyte is believed to have had an impact on the obtained limit of detection in this study. Earlier studies have reported Gly detection down to 1 μM, after extensive chip modification and electrolyte addition to samples [2,19,20]. In this study, we have proved that we are capable of obtaining a Gly signal distinguishable from the background signal, even though no salt or other forms of electrolyte were applied. Based on the obtained signals, it is assumed that the natural content of salts in tap water is enough to carry the current needed for the electrochemical detection of glyphosate. This is proven to be a possibility when looking at Figure 5, where it was possible to detect Gly in a real ground water sample without any pretreatment. Although we have not reached the detection limits of conventional instruments relying on chromatographic techniques, such as high-performance liquid chromatography (HPLC) and gas chromatography that can measure Gly down to 0.1 nM, we have been able to reach the regulation level in the US [21,22]. Further optimisation of the electode needs to be performed in order to achieve a better response and reach the EU regulation requirements. One possible way of enhancing the signal is to test the effect of the working electrode surface area and the surface area ratio between the working and counter electrodes. Another possibile way to enhance the detection limit is to apply molecular imprinted polymers as a recognition layer on the electrodes. This is currently under further investigation.

## 5. Conclusions

This study is a proof-of-concept demonstration of direct Gly detection in drinking water by electrochemical sensing without any sample pretreatment or sensor modification. Although Gly, so far, has been reported as an electrochemically inactive compound, we have demonstrated that Gly indeed can be electrochemically detected, directly, with the correct parameters and electrode material, with a measuring time of only 50 s. The findings of this work are an important step towards lab in the field applications. Future work will need to further investigate more complex water samples containing compounds with a similar structure to Gly, and more ions that could potentially interfere with the signal, before conclusively stating that this method can be applied in the field.

## Supplementary Materials

The following are available online at http://www.mdpi.com/1424-8220/18/9/2961/s1.

## References

[1] Marc J., Mulner-Lorillon O., Bellé R. (2004). Glyphosate-based pesticides affect cell cycle regulation. Biol. Cell.

[2] Habekost A. (2017). Rapid and sensitive spectroelectrochemical and electrochemical detection of glyphosate and AMPA with screen-printed electrodes. Talanta.

[3] Do M.H., Florea A., Farre C., Bonhomme A., Bessueille F., Vocanson F., Tran-Thi N.T., Jaffrezic-Renault N. (2015). Molecularly imprinted polymer-based electrochemical sensor for the sensitive detection of glyphosate herbicide. Int. J. Environ. Anal. Chem..

[4] Moraes F.C., Mascaro L.H., Machado S.A.S., Brett C.M.A. (2010). Direct electrochemical determination of glyphosate at copper phthalocyanine/multiwalled carbon nanotube film electrodes. Electroanalysis.

[5] Mesnage R., Defarge N., Spiroux de Vendômois J., Séralini G.E. (2015). Potential toxic effects of glyphosate and its commercial formulations below regulatory limits. Food Chem. Toxicol..

[6] Pintado S., Montoya M.R., Rodríguez-Amaro R., Mayén M., Mellado J.M.R. (2012). Electrochemical determination of glyphosate in waters using electrogenerated copper ions. Int. J. Electrochem. Sci..

[7] Rogers K.I.M.R., Gerlach C.L. (1996). A Status Report. Comput. Law Secur. Rev..

[8] Wang J. (2002). Real-time electrochemical monitoring: Toward green analytical chemistry. Account. Chem. Res..

[9] Teófilo R.F., Reis E.L., Reis C., da Silva G.A., Paiva J.F., Kubota L.T., da-Silva G.A., Paiva J.F., Kubota L.T. (2007). Glyphosate Determination in Soil, Water and Vegetables Using DPV Optimized by Response Surface Methodology. Port. Electrochim. Acta.

[10] Teófilo R.F., Reis E.L., Reis C., Silva G.A., Kubota L.T. (2004). Experimental Design Employed to Square Wace Voltammetry Response Optimization for the Glyphosate Determination. J. Braz. Chem. Soc..

[11] Pintado S., Amaro R.R., Mayén M., Mellado J.M.R. (2012). Electrochemical determination of the glyphosate metabolite aminomethylphosphonic acid (AMPA) in drinking waters with an electrodeposited copper electrode. Int. J. Electrochem. Sci..

[12] Alatraktchi F.A., Andersen S.B., Johansen H.K., Molin S., Svendsen W.E. (2016). Fast selective detection of pyocyanin using cyclic voltammetry. Sensors.

[13] Lacombe M., Garçon V., Comtat M., Oriol L., Sudre J., Thouron D., Le Bris N., Provost C. (2007). Silicate determination in sea water: Toward a reagentless electrochemical method. Marchem. Chem..

[14] Bian C., Bai Y., Xia S., Tong J., Wang J. (2014). Electrochemical microsensor based on gold nanoparticles modified electrode for total phosphorus determinations in water. IET Nanobiotechnol..

[15] Jońca J., León Fernández V., Thouron D., Paulmier A., Graco M., Garçon V. (2011). Phosphate determination in seawater: Toward an autonomous electrochemical method. Talanta.

[16] Bai Y., Tong J., Bian C., Xia S. An electrochemical microsensor based on molybdophosphate complex for fast determination of total phosphorus in water. Proceedings of the 8th Annual IEEE International Conference on Nano/Micro Engineered and Molecular Systems.

[17] Wang F., Tong J., Li Y., Bian C., Sun J., Xia S. (2014). An electrochemical microsensor based on a AuNPs-modified microband array electrode for phosphate determination in fresh water samples. Sensors.

[18] Horwood E., Southampton Electrochemistry Group (1985). Instrumental Methods in Electrochemistry.

[19] Songa E.A., Somerset V.S., Waryo T., Baker P.G.L., Iwuoha E.I. (2009). Amperometric nanobiosensor for quantitative determination of glyphosate and glufosinate residues in corn samples. Pure Appl. Chem..

[20] Songa E.A., Arotiba O.A., Owino J.H.O., Jahed N., Baker P.G.L., Iwuoha E.I. (2009). Electrochemical detection of glyphosate herbicide using horseradish peroxidase immobilized on sulfonated polymer matrix. Bioelectrochemistry.

[21] Royer A., Beguim S., Tabet J.C., Hulot S., Reding M.A., Communal P.Y. (2000). Determination of Glyphosate and Aminomethylphosphonic Acid Residues in Water by Gas Chromatography with Tandem Mass Spectrometry after Exchange Ion Resin Purification and Derivatization. Application Vegetable Matrixes. Anal. Chem..

[22] Steinborn A., Alder L., Michalski B., Zomer P., Bending P., Martinez S.A., Mol H.G.J., Class T.J., Pinheiro N.C. (2016). Determination of Glyphosate Levels in Breast Milk Samples from Germany by LC-MS/MS and GC-MS/MS. J. Agric. Food Chem..

