# Supplementary material for: Detection of Glyphosate in Drinking Water: A Fast and Direct Detection Method without Sample Pretreatment
Source: Sensors (Basel). 2018 Sep 5;18(9):2961. doi: 10.3390/s18092961 (PMC6163928; doi:10.3390/s18092961)
Supplement: Supplementary file 1 [file sensors-18-02961-s001.pdf]

Supplementary Material

Table S1. Analytical values for available ions in the water sample. Data provided by Mineral Water A/S, Denmark.

Parameter	Value	Units
Calcium	<40	mg/L
Magnesium	<15	mg/L
Sodium	<12	mg/L
Bicarbonates	<150	mg/L
Sulfate	<5	mg/L
Nitrate	<1	mg/L
Potassium	<5	mg/L
Chloride	<13	mg/L
pH	7–7.6	-

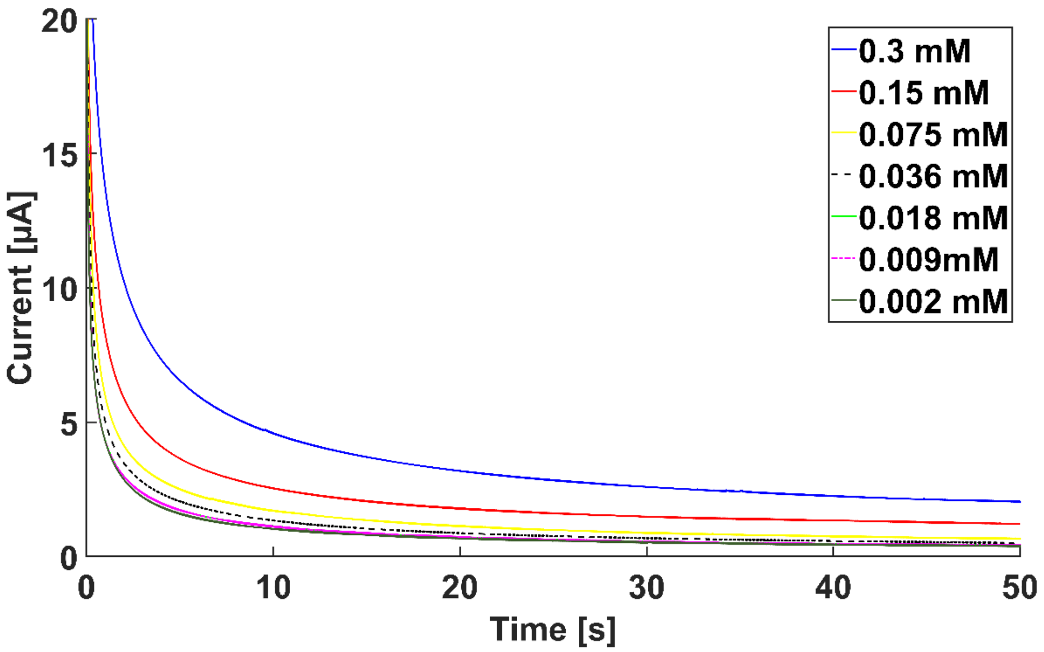

Figure S1. Amperometric measurements using the optimized potential of 0.78 V on different concentrations of glyphosate using a gold working electrode.
